# Rare FN1 missense mutations indicate a protective role against Lewy body dementia in APOEε4 homozygous carriers

**DOI:** 10.1007/s00401-025-02925-z

**Published:** 2025-08-29

**Authors:** Paolo Reho, Anindita Ray, Karri Kaivola, International L. B. D. Genomics Consortium, Badri N. Vardarajan, Haotian Wu, Sonja W. Scholz

**Affiliations:** 1 Neurodegenerative Diseases Research Section, National Institute of Neurological Disorders and Stroke, Bethesda, MD 20892-3707 USA; 2 Department of Environmental Health Sciences, Mailman School of Public Health, Columbia University, New York, NY USA; 3 Translational Immunology, Research Programs Unit, University of Helsinki, Helsinki, Finland; 4 Taub Institute for Research On Alzheimer’s Disease and the Aging Brain, College of Physicians and Surgeons, Columbia University, New York, NY USA; 5 The Gertrude .H. Sergievsky Center, Vagelos College of Physicians and Surgeons, Columbia University, New York, NY USA; 6 Department of Neurology, Johns Hopkins University Medical Center, Baltimore, MD USA

Lewy body dementia (LBD) is the second most common type of neurodegenerative dementia in the aging population. LBD falls under the umbrella of Alzheimer’s disease and related dementias that share some genetic risk factors and clinicopathological features [1, 2]. In a recent study, Bhattarai and collaborators identified a rare variant in *FN1* (c.1070G > A; p.G357E) that protected against Alzheimer’s disease in *APOE* ε4 homozygotes [3]. Thus, we investigated the role of *FN1* mutations in a European ancestry population extracted from the Chia et al. study cohort, consisting of 212 LBD cases and 61 neurologically healthy controls carrying two *APOE* ε4 alleles. We excluded subjects aged ≤ 40 years or those with missing age information (n = 3 controls, n = 1 case) to match the age distribution between cases and controls (Supplementary Table 1, Supplementary Fig. 1 and 2) [1]. Our study cohort included 168 pathologically definite (79.6%) and 43 clinically probable (20.4%) LBD patients diagnosed per consensus criteria [4, 5]. We extracted *FN1* (NM_212482.4) rare missense and loss-of-function (LoF) variants (MAF < 0.01 in the control cohort) annotated with the Ensembl Variant Effect Predictor (v.101). We performed the Optimized Sequence Kernel Association Test (SKAT-O) implemented in the RVtests package (v.2.1.0), including consensus age, sex, and four principal components (PC1, PC3, PC4, PC6) as covariates (Supplementary Materials).

We identified five *FN1* missense substitutions in three (1.4%) LBD cases and five (8.6%) controls (Table 1). Notably, all patients were pathologically diagnosed with definite dementia with Lewy bodies. Of these, two cases also showed Alzheimer’s co-pathology at pathological evaluation. For the remaining cases, information on Alzheimer’s co-pathology was not available (Supplementary Table 2). Interestingly, we detected an enrichment of *FN1* missense mutations in healthy controls compared to LBD patients (SKAT-O *p*-value = 0.008), indicating a protective effect. We did not identify *FN1* LoF variants in our cohort nor rare mutations in known LBD risk genes (*GBA*, *SNCA*, *APP*, *PSEN1*, *PSEN2*) in the *FN1* mutation carriers. Additionally, the analysis of *FN1* rare variants in the *APOE* ε4 heterozygous (ε4/ε3, ε4/ε2) and *APOE* ε4 non-carrier (ε3/ε3, ε3/ε2, ε2/ε2) subgroups revealed a significant enrichment of missense mutations in *APOE* ε4 non-carrier controls compared to LBD cases (Skat-O p-value = 3.32 × 10⁻⁶; Supplementary Table 3). In conclusion, although the small sample size may represent a limitation of our study, we provide supportive evidence for the enrichment of *FN1* rare missense mutations in *APOE* ε4/ε4 and *APOE* ε4 non-carrier healthy controls compared to LBD patients. Our findings corroborate previous evidence suggesting a protective role of *FN1* missense mutations in *APOE* ε4 homozygotes, and extend this evidence to other *APOE* genotypes (Table 1).

**Table 1:** Rare *FN1* missense mutations found in LBD cases and controls

Variant	dbSNP ID	Allele frequency			OR (95% CI)	Fisher’s *p*-value	Variant classification			
		gnomAD	Cases	Controls			ACMG	HGMD	ClinVar	CADD score
c.1139A > G p.Q380R	NA	NA	0	8.62E-03	0	0.21	LB	NA	NA	22.8
c.1775G > A p.R592H	rs147831535	6.59E-03	2.36E-03	8. 62E-03	0.27 (0.02–4.38)	0.38	B	NA	US/LB	27.0
c.1829G > T p.G610V	rs745902139	4.80E-05	0	8. 62E-03	0	0.21	LB	NA	NA	16.9
c.4486C > T p.R1496W	rs139078629	9.02E-03	4.72E-03	8. 62E-03	0.55 (0.05–6.06)	0.52	B	NA	US/LB/B	23.8
c.7274G > A p.R2425H	rs148505961	8.19E-04	0	8. 62E-03	0	0.21	B	NA	LB	4.2

Rare *FN1* missense mutations associated with *APOE* ε4/ε4 LBD cases and neurologically healthy controls (SKAT-O p-value = 0.008)

*OR* odd Ratio, *NA* not applicable/not available, *DM?* possible disease-causing mutation, *US* uncertain significance, *LB* likely benign

## Supplementary Information

Below is the link to the electronic supplementary material.

Supplementary file1 (DOCX 258 KB)

## Data Availability

Genome sequence data for individual LBD patients and resource controls are available at dbGaP (https://www.ncbi.nlm.nih.gov/gap/; accession no. phs001963.v1.p1 NIA DementiaSeq) and at the AMP-PD web portal (https://amp-pd.org).
